# Traumatic Bronchus Avulsion Managed With Extracorporeal Membrane Oxygenation, Pneumonectomy, and Rib Fixation

**DOI:** 10.1155/cris/1079046

**Published:** 2025-12-22

**Authors:** Gillian D. Hertslet, Kaylan N. Gee, Thomas W. Mazonas, Sean A. Jordan, John E. Griepentrog

**Affiliations:** ^1^ DeBusk College of Osteopathic Medicine, Lincoln Memorial University, Knoxville, Tennessee, USA, lmunet.edu; ^2^ Milken Institute School of Public Health, The George Washington University, District of Columbia, Washington, USA, gwu.edu; ^3^ Department of Surgery, University of Tennessee Graduate School of Medicine, Knoxville, Tennessee, USA, tennessee.edu

## Abstract

The mortality of injuries sustained from blunt thoracic trauma (TT) is significantly higher than the mortality of penetrating injuries, and decisions made during the first hour of hospitalization play a critical role in determining outcomes. Patient survival depends on the effective management of injuries that result in the disruption of respiration, circulation, or both. Presented is a case of a 44‐year‐old female who survived a traumatic avulsion of the right lung from the bronchus intermedius, subsequent multifactorial shock, and complications associated with her complex treatment plan, including emergent use of extracorporeal membrane oxygenation (ECMO), completion pneumonectomy, and rib fixation.

## 1. Introduction

Thoracic trauma (TT) accounts for a third of total trauma‐related deaths internationally and is a major contributor to all polytrauma‐related deaths [1, 2]. Blunt trauma is responsible for most thoracic injuries, with the most common cause of blunt TT being motor vehicle collisions (MVCs). Injuries sustained typically result in the disruption of respiratory and/or circulatory function [3]. Those who develop respiratory failure as a result of their injuries require intubation and positive pressure ventilation to correct the hypoxia and hypercarbia. Conventional mechanical ventilation is the main treatment for trauma‐associated hypoxic or hypercapnic respiratory failure. To provide circulatory and respiratory support when all conventional therapeutic methods are unsuccessful, extracorporeal membrane oxygenation (ECMO) can serve as an adjunct therapy to manage the complications associated with post‐traumatic shock [4, 5]. An advanced life support modality for critically ill patients presenting with cardiac arrest and/or respiratory failure, ECMO allows the heart and lungs to “rest” by providing tissue perfusion and oxygenation while permitting reduced ventilator settings to limit further barotrauma [3, 6]. For patients suffering from severe respiratory failure and acute respiratory distress syndrome (ARDS), veno‐venous (VV) ECMO is most appropriate [7]. Veno‐arterial (VA) ECMO is most appropriate for patients suffering from cardiac arrest and cardiogenic shock [8–10]. Clinical outcomes for ECMO use in adult trauma patients who suffered shock and ARDS are highly uncertain due to unknown adverse complications. Use in trauma scenarios is extremely controversial as most studies are retrospective or registry in nature, providing no clear guideline on the timing of its administration or initiation [4, 7, 11].

To our knowledge, this represents a rare survival case owing to the novel use of multiple modalities not typically leveraged in trauma scenarios.

## 2. Case Presentation

**Figure 1:**
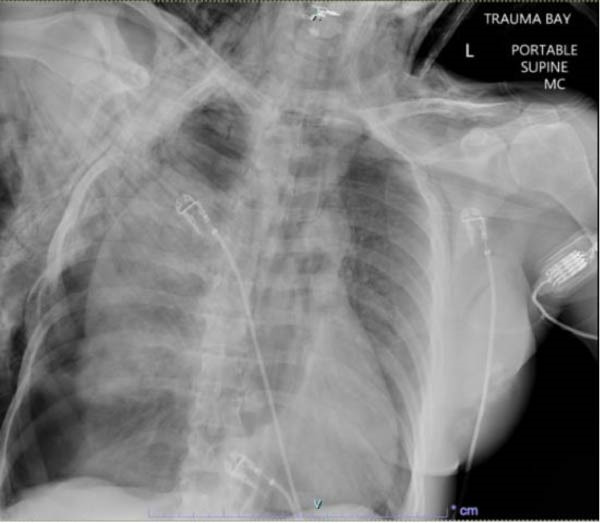
Initial chest radiography on arrival in trauma bay. A radiograph potentially shows the “fallen lung sign,” a rare but specific indication of tracheobronchial injury in the case of persistent pneumothorax.

**Figure 2:**
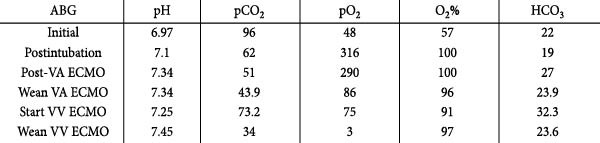
Breakdown of arterial blood gas on arrival at the ED, postintubation, post‐VA ECMO cannulation, weaning from VA ECMO, starting VV ECMO, and weaning from VV ECMO.

**Figure 3:**
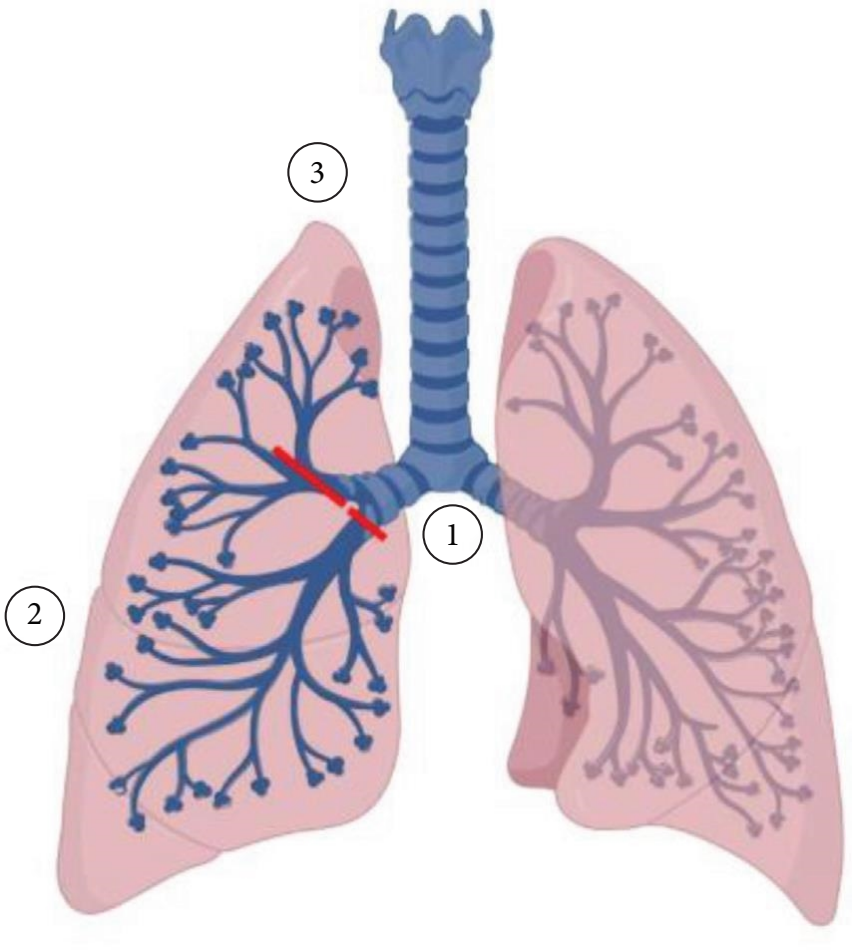
Model of traumatic bronchial avulsions. (1) Complete avulsion of the right bronchus intermedius from the right mainstem bronchus. (2) Completely avulsed right lower and middle lobe segments due to (1). (3) Almost complete avulsion of the right upper lobe; anterior and posterior segments completely avulsed. Only the apical segment intact.

A 44‐year‐old female presented to the emergency department after a MVC versus pedestrian, which resulted in a significant crush injury to the chest. On arrival, the patient had a Glasgow Coma Scale (GCS) of 15 and an initial oxygen saturation (SpO_2_) of 70%. The patient was tachycardic with stable blood pressure. Significant crepitus in the chest and decreased breath sounds on the right side were present. Chest X‐ray demonstrated a right pneumothorax for which a tube thoracostomy was performed with minimal blood return and was ultimately unsuccessful in improving oxygenation, with spO_2_ levels decreasing to 57% on the high‐flow nonrebreather (Figures 1 and 2). Arterial blood gas (ABG) demonstrated profound respiratory acidosis (Figure 2). The patient was subsequently intubated for acute hypoxic respiratory failure. A continuous air leak from the chest tube was present and persisted after suction was discontinued. A left tube thoracostomy was performed with no effect on spO_2_. The patient had low‐end tidal carbon dioxide (etCO_2_) despite intact circulatory function and remained tachycardic with stable blood pressure. External examination and a focused assessment with sonography for trauma (FAST) exam were performed with no identifiable source of bleeding. Bronchoscopy demonstrated complete avulsion of the right upper lobe anterior and posterior segments, with the apical segment appearing to be intact, accompanied by significant trauma to the bronchus intermedius with an inability to identify the right middle and lower lobe segments due to significant extravasation blocking the visual field (Figure 3). The endotracheal tube was adjusted to terminate in the left mainstem bronchus but was unsuccessful in resolving the patient’s hypoventilation (Figure 2). Due to the inability to manage persistent respiratory derangements resulting from the patient’s extensive right thoracic and pulmonary injuries, multidisciplinary discussion between trauma, cardiothoracic, and ECMO teams was held, resulting in the decision to start femoral–femoral VV ECMO for respiratory support.

While the ECMO team assembled, completion imaging was performed to assess traumatic injuries and rule out intracranial bleeding (Figure 4). No contraindications to ECMO cannulation were noted. During the initial cannulation attempt, the patient went into cardiac arrest. Cardiopulmonary resuscitation (CPR) was initiated, and the decision was made to place the patient on VA ECMO for cardiac support. A previous arterial line in the right femoral artery was replaced with a 17 French arterial cannula. A previous right femoral venous line was replaced with a 25 French venous drainage cannula. The patient was then admitted to the ICU in critical condition for further resuscitation.

Figure 4Axial (a) and coronal (b) views of computed tomography (CT) immediately post‐trauma. Obvious avulsion of the right lung from the bronchus, multiple displaced rib fractures resulting in flail chest, and severe hemopneumothorax with significant mediastinal shift.

**Figure:**
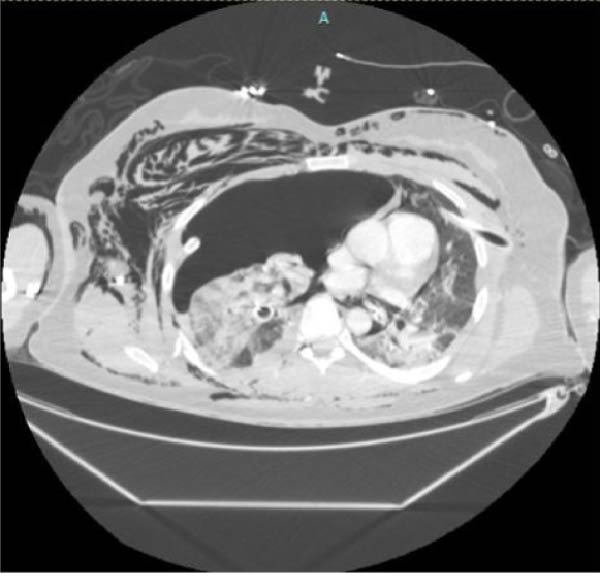
(a)

**Figure:**
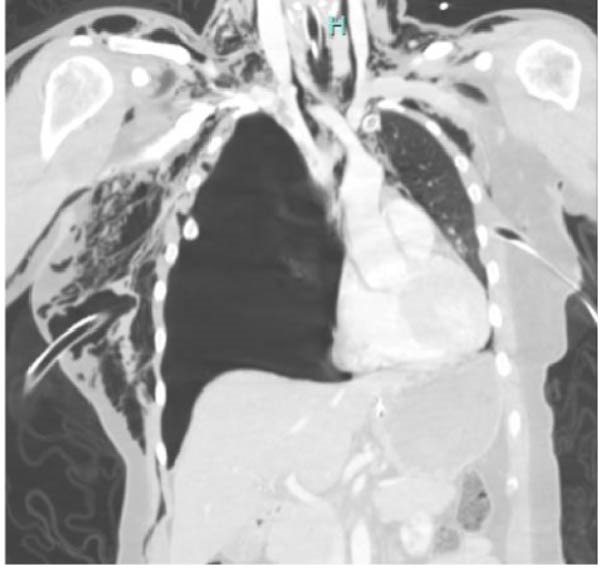
(b)

**Figure 5:**
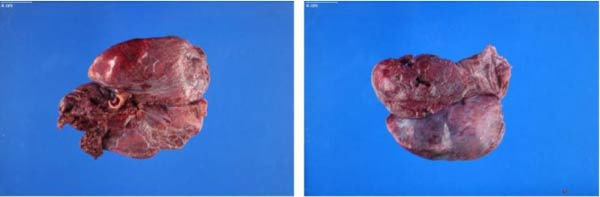
A 540 g right lung postcompletion pneumonectomy due to traumatic right lung avulsion from the bronchus intermedius. Multiple hemorrhagic lacerations are present, and the bronchus is transected with irregular, torn‐appearing borders. The lung tissue was boggy, heavy, and hemorrhagic. Nonviable due to the multiple traumatic injuries.

**Figure 6:**
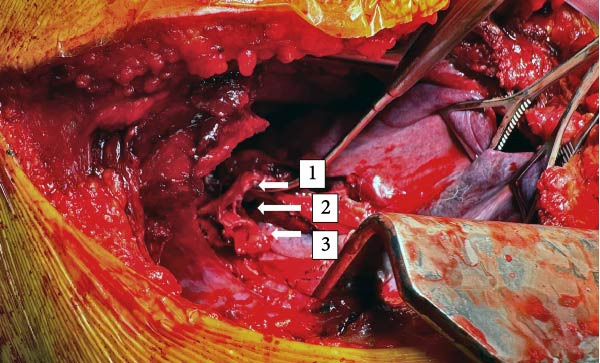
Intraoperative photo of irreparable shredded bronchus. (1) Right upper lobe bronchus. (2) Injury at secondary carina. (3) Avulsed bronchus intermedius.

Due to massive transfusion requirements, the right pneumonectomy was performed on hospital day one (Figures 5 and 6). Secondary to failure to wean from VA ECMO on day two, a ventricular assist device (Impella 5.5) was positioned to support ventricular unloading. On day 3, the patient experienced menorrhagia requiring multiple blood transfusions and underwent uterine artery embolization with interventional radiology. On day 5, the patient was decannulated from VA ECMO. An echocardiogram performed on day 6 demonstrated moderate pulmonary hypertension, for which epoprostenol was started. That night, an ABG demonstrated increasing respiratory acidosis (pH 7.252, pCO2 73.2 mmHg, pO2 75, spO_2_ 91%, HCO_3_ 32.3). Nebulized epoprostenol was unsuccessful in alleviating pulmonary hypertension; pulmonary artery pressure remained at 80–90 mmHg. Thus, the decision was made to proceed with VV ECMO via right internal jugular 30 French dual lumen cannulation to provide respiratory support. On day 9, the patient underwent tracheostomy and open reduction and internal fixation (ORIF) of segmentally fractured right ribs 4–7 (Figure 7). Reducing the flail chest and stabilizing the chest wall improved the patient’s chest wall mechanics.

Figure 7Intraoperative photos before (a) and after (b) ORIF of right ribs 4–7.

**Figure:**
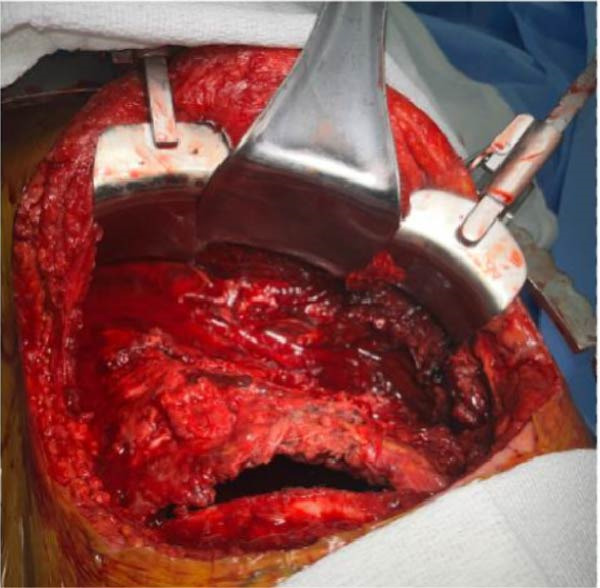
(a)

**Figure:**
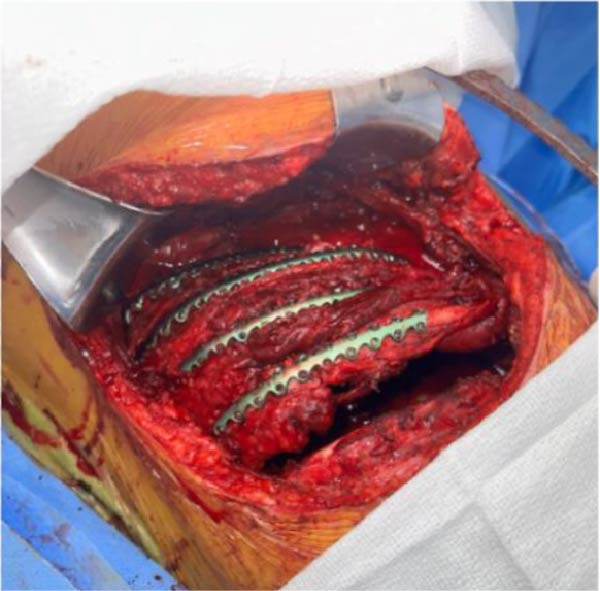
(b)

**Figure 8:**
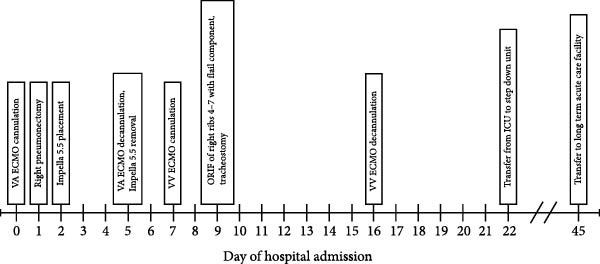
Timeline of patient hospital stay and associated milestones.

Focus shifted to repairing the patient’s remaining non‐fatal orthopedic injuries and managing her postoperative pulmonary edema, pulmonary hypertension, and right‐sided heart failure. Pulmonary hypertension was managed with IV epoprostenol and sildenafil, while edema was removed by ultrafiltration of the ECMO circuit. The patient had been on minimal ECMO support (FiO2 0.35, PEEP 6) since day 11 and was tolerating capping trials. After tolerating 24 h of tracheostomy capping, the patient was decannulated from VV ECMO on day 16. On day 21, a repeat echocardiogram showed improvement of right ventricular function, which allowed the patient to be weaned from epoprostenol and sildenafil. The patient was transferred to the step‐down unit on day 22 of her hospital stay and was discharged from the hospital to a long‐term acute care facility on day 45 (Figure 8).

## 3. Discussion

This case represents a rare and unique survival scenario in trauma due to the combined utilization of VA and VV ECMO and a ventricular assist device for salvage cardiopulmonary support, survival following trauma pneumonectomy, and restoration of anatomic integrity of the chest wall with rib fixation.

The “fallen lung sign” is a radiographic clue of main bronchial avulsion and is characterized by a collapsed lung that falls into a dependent position and remains attached only by the hilar vasculature. Although rare, this sign is highly specific for tracheobronchial injury in the setting of trauma and is helpful in cases of unexplained persistent pneumothorax [12, 13]. In our case, the fallen lung sign was not identified in the initial imaging interpretation due to a lack of familiarity with this finding at the time. This omission does not affect the validity of the diagnosis, though initial recognition of the fallen lung sign would have provided an additional supportive radiographic feature.

### 3.1. ECMO

Utilization of ECMO is an increasingly valuable tool in the management of TT with severe disruption of the cardiopulmonary system and has been reported with increasing frequency as a viable option in the management of severely injured trauma patients who suffer from multifactorial shock and respiratory failure [10, 14–16]. Nevertheless, clear guidelines regarding the use of ECMO in trauma patients, including the full scope of associated complications, are lacking, and thus the decision to pursue ECMO in the trauma setting should be highly individualized [17, 18]. VV ECMO as a nonventilatory strategy for severe hypoxemic respiratory failure mitigates the vicious cycle of pulmonary dysfunction and barotrauma associated with lung injury due to prolonged and elevated inspiratory pressures, particularly in patients suffering from ARDS [16].

Patients requiring multiple ECMO runs are known to have high mortality rates: survival declines from 36.7% for two runs to 29.4% for three or more runs. Mortality is worse when cardiac support is required on the first run, rather than pulmonary support alone [4, 19–21]. Yet, in the case presented, two separate ECMO cannulations with distinct configurations were employed, and the patient recovered. Persistent hypoxemia and devastating right lung injury prompted a plan for VV ECMO, which was quickly escalated to VA ECMO after cardiac arrest.

What further sets this case apart is the innovative use of a percutaneous ventricular assist device in conjunction with VA ECMO to offload the left ventricle (LV), a strategy seldom described in trauma literature. This device supported left ventricular function against the increased afterload commonly seen in VA ECMO and allowed for early assessment of residual global myocardial function [22, 23]. Isath et al. [24] conducted a study that supports the utilization of Impella 5.5 due to the optimization of recovery and the capacity to enable mobility in patients who suffered cardiogenic shock.

While VA ECMO assisted in hemodynamic stabilization and allowed for definitive surgical intervention for the patient’s devastating pulmonary injuries, hemorrhage on ECMO was a primary concern, necessitating adoption of a heparin‐minimized protocol [11, 18]. Menorrhagia on day 3 was successfully managed percutaneously by interventional radiology, and there were no further hemorrhagic complications.

The average VA ECMO duration is 3–4 days [5]. When weaning from VA ECMO, parameters such as a left ventricular ejection fraction greater than 25% and a normal cardiac index >2.5 L/min indicate cardiac recovery [25]. As vasopressors and ECMO support were weaned, transesophageal echocardiogram demonstrated improved left ventricular function with an ejection fraction of 60%–65% (previously 25% on day 2). Cardiac function had recovered sufficiently for decannulation from VA ECMO on day 5.

Post‐pneumonectomy, pulmonary hypertension, and pulmonary edema contributed to the patient’s worsening hypoxic respiratory failure. A pulmonary artery pressure of 65/31 mmHg, a decrease in spO2 (43%), increasing respiratory support (FiO2 100%, PEEP 8), and epoprostenol requirements necessitated re‐cannulation with VV ECMO on day 7. It could be argued that the initial decannulation from VA ECMO may have been too early. However, cardiac function had returned, and the patient met the criteria for decannulation on day 5. Re‐cannulation with VV ECMO was most appropriate in this case to provide pulmonary support and allow for further organ recovery postpneumonectomy [19].

### 3.2. Pneumonectomy

Initial bronchoscopy in the trauma bay revealed what appeared to be a complete avulsion of the bronchus intermedius and a near‐complete avulsion of the right upper lobe airway (Figure 3). Persistent hemodynamic instability from the patient’s injuries increased transfusion requirements throughout the first night. The first glance of the thoracic cavity in the OR demonstrated significant damage to the vasculature that was beyond repair (Figures 5 and 6), and a completion pneumonectomy was warranted to manage intrathoracic bleeding from extensive bronchial disruption [26]. The high morbidity and mortality associated with pneumonectomy independent of trauma scenarios necessitates presurgical testing but was clearly forgone due to the emergent nature of this case. Less than 1% of patients suffering from TT will require a pneumonectomy, which is more commonly performed for blunt pulmonary injuries (36% blunt compared to 23% penetrating) [27]. When performed for treatment of traumatic lung injuries, pneumonectomy is typically fatal, with mortality rates ranging from 50% to 100% [26, 28]. Postoperative pulmonary edema, pulmonary hypertension, and right ventricular failure are commonly noted, occurring in 2%–5% of cases [26, 29, 30]. The rapid increase in pulmonary artery pressure prevents the thin‐walled right ventricle from pumping effectively and increases the risk of mortality by 50% [26, 29]. Supportive care with ventilatory support and judicious fluid resuscitation to target euvolemia is key. Ultrafiltration through the ECMO circuit was employed to curtail pulmonary edema, and pulmonary hypertension was treated with epoprostenol and sildenafil.

### 3.3. Rib Fixation

Surgical stabilization of segmental, displaced rib fractures was deemed necessary to restore chest wall stability and assist in weaning from VV ECMO. Rib fractures, commonly seen after blunt TT and CPR, are traditionally managed nonoperatively with pain control, pulmonary hygiene, and immediate mobilization [31]. Chest wall instability secondary to rib fractures can significantly impair respiratory mechanics and hinder mechanical ventilation weaning strategies [32]. For patients with flail chest and respiratory failure, rib fixation is indicated over nonoperative management due to the reduction in mortality and decreased incidence of pneumonia [31].

## 4. Conclusion

This is a rare use of both VA and VV ECMO in a traumatic bronchus avulsion to manage the subsequent multifactorial shock. Utilizing VA ECMO early for management of shock and hemodynamic instability was a time‐sensitive decision that was crucial in saving the patient’s life. Pneumonectomy successfully controlled continued intrathoracic bleeding and resultant hemorrhagic shock. Employing VV ECMO for respiratory support allowed for further organ recovery and addressed postoperative pulmonary edema, pulmonary hypertension, and right heart failure. Rib fixation successfully reduced the flail chest, improving breathing mechanics and chest wall stability. In summary, this case’s novelty lies in the infrequent survival after multiple sequential ECMO runs, combining both VV and VA ECMO modalities, the integration of a ventricular assist device for left ventricular unloading during VA ECMO, and successful management of post‐pneumonectomy respiratory failure with rib fixation—all of which illustrate an aggressive but effective multidisciplinary approach with contributions from the trauma, cardiothoracic, and ECMO teams to achieve a favorable outcome for a complex polytrauma patient with profound cardiopulmonary compromise.

### 4.1. Clinical Pearls

• A multidisciplinary approach (including the use of VV and VA ECMO, pneumonectomy, and rib fixation) to support the patient through deranged physiology and restore chest wall mechanics helped improve the outcome of this patient.
• Utilization of both VV and VA ECMO was necessary to improve hemodynamic instability and allow for the management of multifactorial shock. Patients on VA ECMO may require additional ventricular support (in this case, Impella 5.5) to decrease left ventricular strain.
• Patients with traumatic bronchial disruption may require pneumonectomy, which puts them at increased risk of pulmonary hypertension, pulmonary edema, and right heart failure.
• Surgical stabilization of an unstable chest wall can improve chest wall mechanics and improve outcomes in complex polytrauma patients.

**Nomenclature**
TT: Thoracic trauma
MVC: Motor vehicle collision
ECMO: Extracorporeal membrane oxygenation
ARDS: Acute respiratory distress syndrome
VV: Veno‐venous
VA: Veno‐arterial
GCS: Glasgow Coma Scale
SpO_2_: Oxygen saturation
FiO_2_: Fraction of inspired oxygen
ABG: Arterial blood gas
EtCO_2_: End tidal carbon dioxide
FAST: Focused assessment with sonography for trauma
CPR: Cardiopulmonary resuscitation
ORIF: Open reduction and internal fixation.

## Consent

Written and verbal consent was obtained from the patient to submit this case report.

## Disclosure

All authors reviewed and approved the final manuscript.

## Conflicts of Interest

The authors declare no conflicts of interest.

## Funding

No funding was received for this manuscript.

## Data Availability

The patient’s clinical data used to support the findings of this study are included within the article.
